# Supplementary material for: Mixed Electronic-Ionic Conductivity and Stability of Spark Plasma Sintered Graphene-Augmented Alumina Nanofibres Doped Yttria Stabilized Zirconia GAlN/YSZ Composites
Source: Materials (Basel). 2023 Jan 9;16(2):618. doi: 10.3390/ma16020618 (PMC9861201; doi:10.3390/ma16020618)
Supplement: Supplementary file 1 [file materials-16-00618-s001.zip › materials-2106850-supplementary.pdf]

Supplementary material

Typical spectra collected from the surface of 0GAlN and 5GAlN composites are shown in Figure S1. Only signal from the free carbon is seen in spectra of both samples (black graphs in Figure 4). Polishing results in the signals from YSZ matrix in the spectra. The signals from graphene or any carbon phase are not seen. Latter points on the lack of GAlN in the near-edge layer. The only exception is the GAlN5 composite with 5 wt.% GAlN addition. For the composite with the highest GAlN content the spectrum collected from the surface after polishing is equal to the one collected from the cross-section.

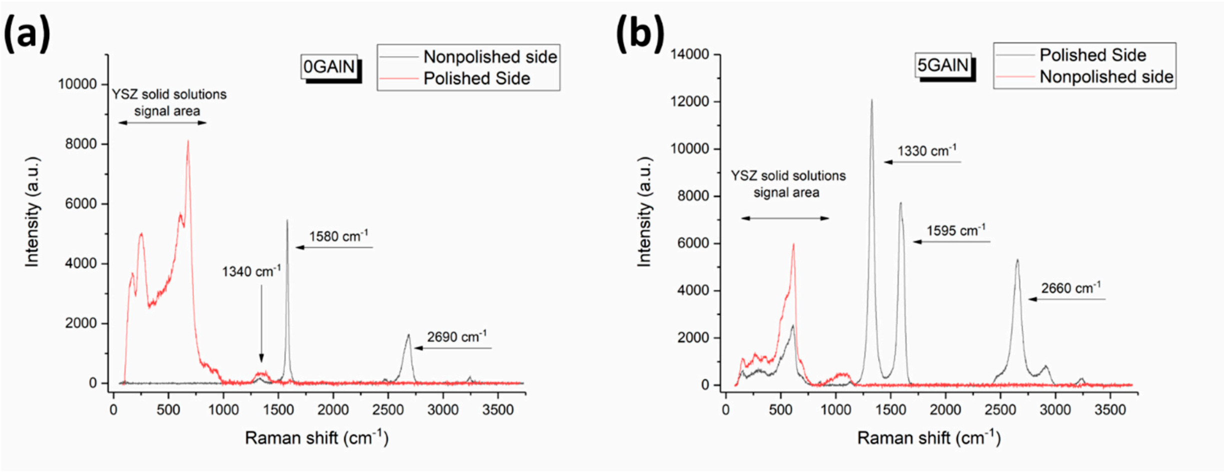

Figure S1. Raman spectra collected from the both polished and non-polished surface of the samples (**a**) 0GAlN and (**b**) 5GAlN.

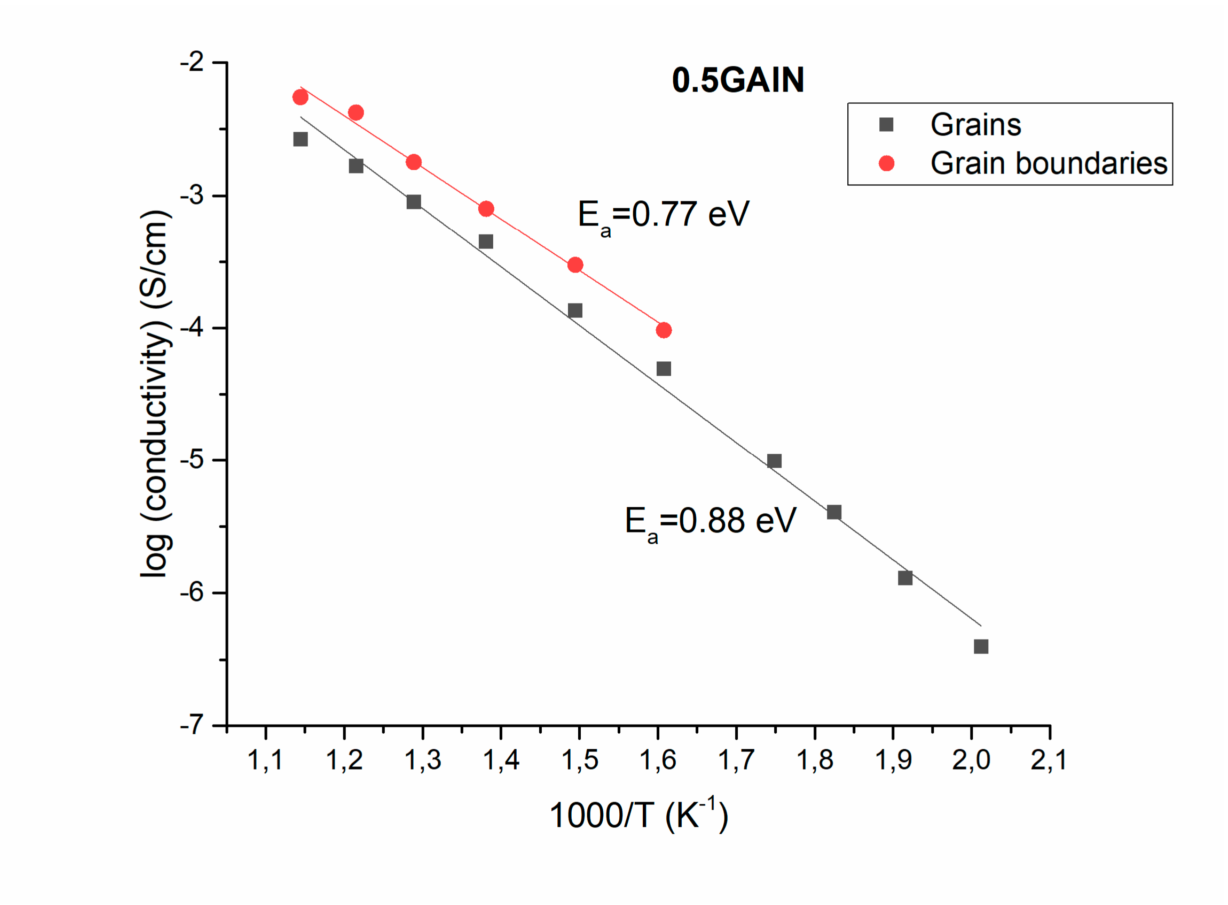

Figure S2. The Arrhenius plots for grain and grain boundary components of conductivity in 0.5GAlN composite.

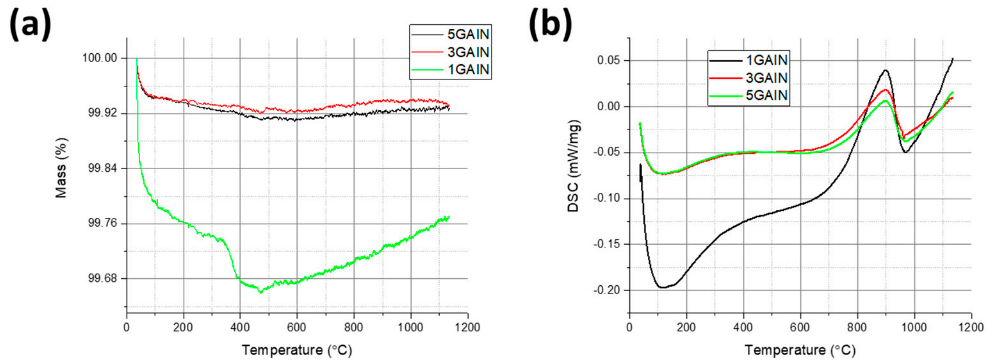

Figure S3. (**a**) Thermogravimetric analysis and (**b**) differential scanning calorimetry results for ceramics 1GAlN-3GAlN.
